# Ultrasound combined with serological markers for predicting neonatal necrotizing enterocolitis: a machine learning approach

**DOI:** 10.3389/fped.2025.1606571

**Published:** 2025-07-14

**Authors:** Yi Yang, Shoulan Zhou, Xiaomin Liu, Yanhong Zhang, Liping Lin, Chenhan Zheng, Xiaohong Zhong

**Affiliations:** Department of Ultrasound, Women and Children's Hospital, School of Medicine, Xiamen University, Xiamen, Fujian, China

**Keywords:** necrotizing enterocolitis, ultrasound, serological markers, machine learning, SHAP values necrotizing enterocolitis, SHAP values

## Abstract

**Background & aims:**

Neonatal necrotizing enterocolitis (NEC) remains a leading cause of morbidity and mortality in preterm infants. Current diagnostic methods, relying on clinical signs and radiography, often lack sensitivity for early detection. This study aimed to develop and validate a machine learning (ML) model integrating ultrasound and serological markers to improve NEC prediction in neonates.

**Methods:**

This retrospective, case-control study included 191 neonates (cases with Bell's stage ≥ II NEC and matched controls) admitted to a tertiary NICU. Data were extracted from electronic medical records, including demographics, clinical variables, ultrasound findings (bowel wall thickness, edema, gas location, peristalsis, seroperitoneum), and serological markers (WBC, neutrophil count, CRP, ALP, albumin, procalcitonin, platelet count, INR, hemoglobin). Twelve ML algorithms were evaluated using 10-fold cross-validation on a training set (70%). The optimal model was selected based on AUC-ROC and further optimized via hyperparameter tuning. Model performance was assessed on an independent validation set (30%). Explainable AI (XAI) using SHAP values was employed to identify key predictive features.

**Results:**

XGBoost demonstrated the highest performance (AUC = 0.97, 95% CI: 0.92–0.99) during cross-validation. The optimized XGBoost fusion model—Ultrasound combined Serological Predict NEC (USPN) achieved an AUC of 0.88 (95% CI: 0.76–0.99) in the validation set, with a sensitivity of 0.73 and specificity of 1.00. The USPN model outperformed models based solely on ultrasound (AUC = 0.73) or serological markers (AUC = 0.79). SHAP analysis identified bowel peristalsis, C-reactive protein, albumin, bowel thickness, and procalcitonin as the most influential predictors. Decision curve analysis demonstrated a positive relative net benefit of the USPN model compared to the US and serological models in the validation set.

**Conclusion:**

A machine learning model integrating ultrasound and serological markers significantly improves the prediction of NEC in neonates compared to single-modality approaches. This multimodal approach has the potential to facilitate earlier diagnosis and intervention, potentially improving outcomes in this high-risk population.

## 1 Introduction

Neonatal necrotizing enterocolitis (NEC) remains a devastating gastrointestinal emergency in neonates, particularly affecting preterm infants with birth weights below 1,500 g (1, 2). Current diagnostic reliance on Bell staging criteria and abdominal radiography faces critical limitations, including delayed detection of early pathophysiological changes (e.g., mucosal ischemia and bacterial translocation) (3, 4). x-ray is an important diagnostic modality for NEC, but its widespread use is limited by radiation concerns and the inconvenience of requiring transport to a radiology suite, rather than being readily available at the bedside. This diagnostic latency contributes to the persistent 20%–30% mortality rate despite advances in neonatal intensive care (5, 6).

Recent advancements in ultrasonography have demonstrated superior sensitivity in detecting preclinical NEC manifestations. High-resolution ultrasound can quantify bowel wall thickness (BWT) variations (>2.0 mm predictive of necrosis) and monitor mesenteric blood flow dynamics through Doppler indices (7, 8). Abdominal radiography remains a common initial imaging modality for evaluating neonatal abdominal pathology. However, as demonstrated by Silva et al. (2013), a normal radiographic gas pattern does not exclude the presence of significant intestinal abnormalities detectable by ultrasound, highlighting the potential for missed diagnoses when relying solely on radiography (9). Parallel developments in serum biomarkers, including PT, INR, APTT (10), C-reactive protein (11) and other serological have shown potential for risk stratification, Sharif demonstrated that low serum albumin (SA) concentration (≤20 g/L) on day 2 of NEC diagnosis is a significant predictor of surgical intervention in neonates with Bell's stage 2 NEC. This finding suggests that SA, in conjunction with other clinical and serological markers, may be a useful tool for identifying patients at higher risk of requiring surgery (12). Their findings suggest that monitoring coagulation parameters can aid in early identification of high-risk NEC neonates, potentially optimizing treatment strategies and improving outcomes (13, 14). While ultrasonography provides real-time visualization of intestinal dynamics, its diagnostic accuracy may be compromised by acoustic shadowing from bony structures and operator-dependent expertise, potentially leading to misinterpretation of early NEC signs. Conversely, serological biomarkers, though objective in quantification, exhibit significant interindividual variability due to fluctuations in host immune status and inflammatory cascades. These inherent limitations of standalone modalities underscore the suboptimal predictive performance when employing either approach in isolation. Emerging evidence suggests that integrating both modalities through machine learning algorithms may harness their synergistic diagnostic potential, thereby improving sensitivity for preclinical NEC detection and risk stratification.

Machine learning (ML) presents transformative opportunities for NEC prediction through multimodal data fusion. Leiva et al. (2023) provide a comprehensive overview of the use of machine learning in NEC biomarker discovery, while also acknowledging the challenges inherent in the field. They highlight the potential of machine learning to integrate multi-omics data with clinical features, phenotypes of progression, and predicted therapeutic targets, resulting in clinically meaningful information. This approach could lead to earlier diagnosis, more targeted therapies, and improved outcomes for infants with NEC (15). Contemporary studies further highlight ML's capacity to decode nonlinear interactions between temporal ultrasound features and biochemical trajectories (16). Our study innovatively expands this paradigm by systematically evaluating 12 ML algorithms on hybrid ultrasound-serological datasets, addressing critical gaps in neonatal predictive modeling.

## 2 Materials and methods

### 2.1 Study design and patient population

This retrospective, case-control study was conducted at Women and Children's Hospital, School of Medicine, Xiamen University, a tertiary neonatal intensive care unit (NICU), between November 2019 and November 2024. The study protocol was approved by the Institutional Review Board (IRB) of Women and Children's Hospital [IRB approval number: (KY-2025-046-K01)], written informed consent was obtained from the parents or legal guardians of all participating infants. All procedures were performed in accordance with the ethical standards of the responsible committee on human experimentation (institutional and national) and with the Helsinki Declaration of 1975, as revised in 2008.

**Figure 1:**
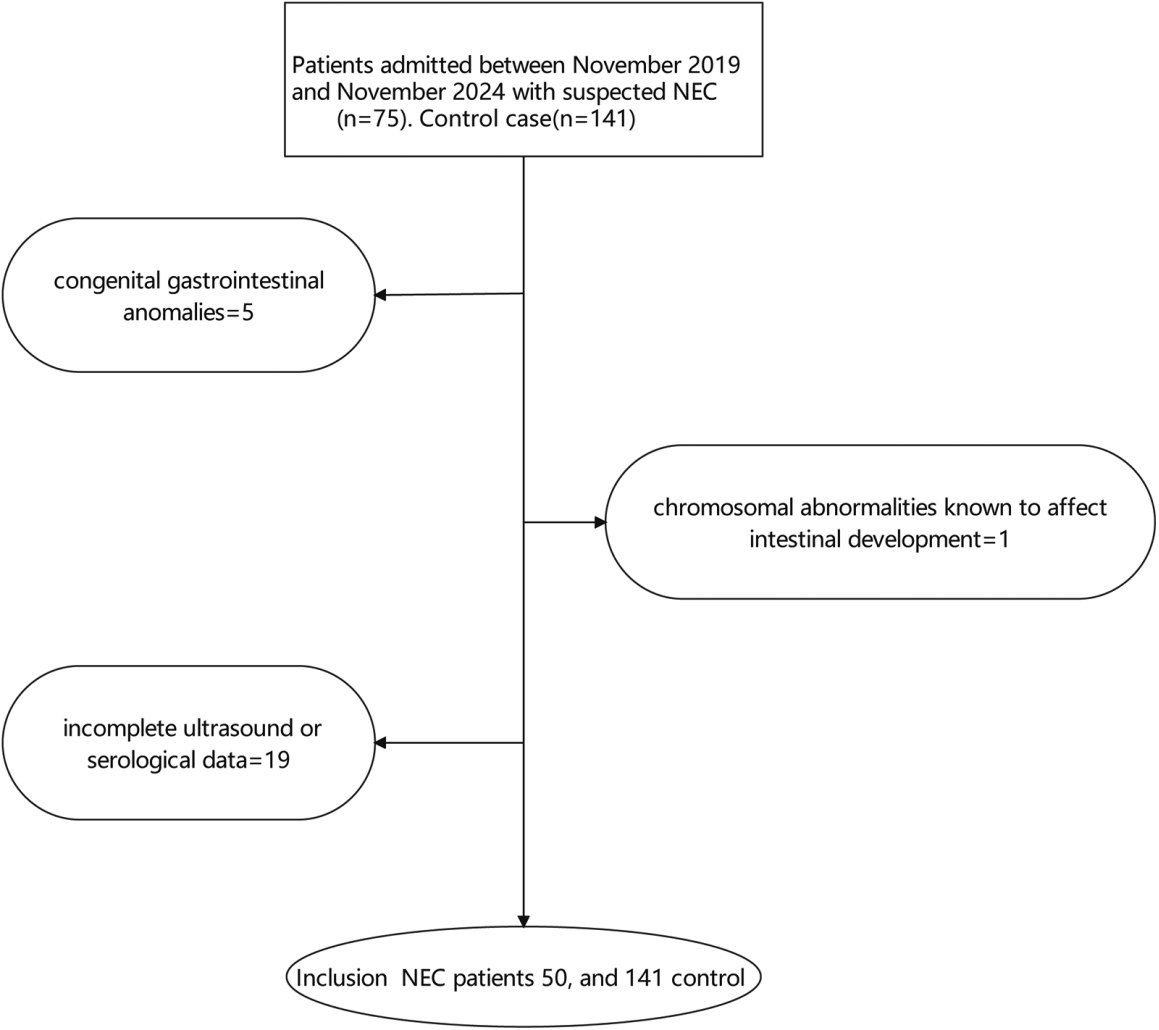
Patients inclusion flow.

NEC diagnosis was based on modified Bell's staging criteria (3). Cases were defined as neonates with Bell's stage ≥ II. Controls were selected from neonates admitted to the NICU during the same period who did not develop NEC and were matched to cases based on gestational age (± 2 weeks) and birth weight (± 200 grams). Exclusion criteria included: (1) congenital gastrointestinal anomalies, (2) chromosomal abnormalities known to affect intestinal development, and (3) incomplete ultrasound or serological data, show as Figure 1.

### 2.2 Data collection

Data were extracted from electronic medical records (EMRs). The following variables were collected for each patient:

Demographic Data: Age (days), Gestational age (gestational): Gestational age at birth (weeks), Sex, Polyembryony: Presence of multiple gestation (yes/no), Birth weight (weight): Birth weight (grams).
Clinical Data: Onset day: Age at onset of symptoms (days), OB: Occult blood in stool (positive/negative), Transfusion: History of blood transfusion (yes/no), Ventilation: Use of mechanical ventilation (yes/no), Antibiotic: Use of antibiotics (yes/no), NRDS: Neonatal respiratory distress syndrome (yes/no), PDA: Patent ductus arteriosus (yes/no), Distress: Intrauterine distress (yes/no), Dirty: Turbid amniotic fluid (yes/no), Embryolemma: Premature rupture of membranes (yes/no), Delivary: Mode of delivery (eutocia, cesarean section), Fetal heart: Abnormal fetal heart rate (yes/no), Mother diabetes: Maternal diabetes (yes/no), Mother HBP: Maternal hypertension (yes/no), Placental inflammation: Placental inflammation (yes/no).
Ultrasound Data: All abdominal ultrasound examinations performed within 24 h prior to NEC diagnosis (for cases) or a randomly selected ultrasound examination during the same period of hospitalization (for controls) were reviewed. These measurements were recorded prior to the clinical diagnosis and before any NEC-specific intervention, ensuring that the variables reflect pre-onset clinical status suitable for predictive modeling. In clinical practice, these assessments were typically performed in response to early, non-specific symptoms (e.g., feeding intolerance, abdominal distension), before a formal NEC diagnosis was made. Thus, the measurements reflect real-world subclinical evaluation rather than post-diagnostic management. The following parameters were extracted: Bowel thickness: Bowel wall thickness at the most affected segment (mm), Bowel edema: Bowel wall edema (yes/no), Gas: Presence and gas (yes/no), Bowel peristalsis: Bowel wall peristalsis (decreased, normal), Seroperitoneum: Presence of free intraperitoneal fluid (yes/no).
Serological Data: Serum levels of the following biomarkers, measured within 24 h prior to NEC diagnosis (for cases) or at the time of the matched ultrasound examination (for controls): Wbc: White blood cell count (×10^9^/L), nec_A: Neutrophil count (%), Crp: C-reactive protein (mg/L), Alp: Alkaline phosphatase (U/L), Alb: Albumin (g/L), Procalcitonin: Procalcitonin (ng/ml), Plt: Platelet count (×10^9^/L), INR: International normalized ratio, WBC_A: Absolute white blood cell count (×10^9^/L), Hgb: Hemoglobin (g/dl). Hgb: Hemoglobin at admission (g/dl), Age: mother's age (years).

### 2.3 Ultrasound image analysis

All ultrasound images were examined by two experienced pediatric radiologists blinded to the clinical outcomes. In cases of disagreement, a third senior physician was consulted for discussion to reach a final decision. Bowel thickness was measured at the thickest point of the bowel wall, perpendicular to the lumen. Bowel peristalsis was graded as decreased, normal, or increased based on visual assessment.

### 2.4 Machine learning model development

The dataset was randomly split into training (70%) and validation (30%) sets. The training set was used to train and optimize the machine learning models, while the validation set was used to evaluate their performance.

We evaluated the performance of various machine learning algorithm, including Logistic Regression (LR), Random Forest (RF), Gradient Boosting (GB), Support Vector Classifier (SVC), Decision Tree, K-Nearest Neighbors (KNN), Naive Bayes, Extreme Gradient Boosting (XGBoost), Light Gradient Boosting Machine (LightGBM), Ridge Classifier, Extra Trees, Adaptive Boosting (AdaBoost), and Voting Classifier.

Model selection was based on performance metrics on the total set using 10-fold cross-validation. The following metrics were used: area under the receiver operating characteristic curve (AUC-ROC), sensitivity, specificity, positive predictive value (PPV), and negative predictive value (NPV). The algorithm with the highest AUC-ROC was selected as the optimal model.

### 2.5 Model training and optimization

The optimal machine learning algorithm was further optimized using hyperparameter tuning via grid search with cross-validation.

### 2.6 Model evaluation

The performance of the optimized model was evaluated on the training set and independent validation set. The following performance metrics were calculated: AUC-ROC, sensitivity, specificity, PPV, NPV, accuracy, Brier score. Calibration curves were generated to assess the model's ability to accurately predict probabilities.

### 2.7 Model comparison

To assess the incremental value of combining ultrasound and serological data, we compared the performance of the following models:

• **Fusion Model:** The optimized model trained on the combined dataset of ultrasound and serological variables, named Ultrasound combined Serological Predict NEC (USPN).
• **Ultrasound Model:** The optimized model trained only on ultrasound variables, named US model.
• **Serological Model:** The optimized model trained only on serological variables, named Serological model.

Model performance was compared using DeLong's test for AUC-ROC differences.

### 2.8 Statistical analysis

Continuous variables were expressed as mean ± standard deviation. Categorical variables were expressed as percentages. Differences between groups were assessed using t-tests or Mann–Whitney *U* tests for continuous variables and chi-square tests or Fisher's exact tests for categorical variables. To assess the calibration of the predictive models, we employed calibration curves. We evaluated calibration using the following metrics: Brier Score: The Brier score measures the mean squared difference between the predicted probabilities and the actual outcomes (0 or 1). It ranges from 0–1, with lower values indicating better calibration. Hosmer-Lemeshow (HL) Test: The HL test is a goodness-of-fit test that assesses the agreement between predicted and observed event rates across groups (typically deciles) of predicted probabilities. A non-significant *p*-value (typically > 0.05) suggests good calibration, indicating no significant difference between predicted and observed event rates. Calibration Slope and Intercept: We performed a logistic regression of the observed outcomes on the predicted probabilities. The calibration slope reflects the spread of predicted probabilities; a slope of 1 indicates ideal calibration. The calibration intercept reflects the average predicted probability when the observed outcome is 0; an intercept of 0 is ideal. Decision curve analysis (DCA) was performed to evaluate the clinical utility of the USPN model compared to models based solely on ultrasound or serological markers. The net benefit was calculated across a range of threshold probabilities, and the relative net benefit (RNB) was derived to quantify the incremental benefit of the USPN model. Additionally, the net reclassification improvement (NRI) and integrated discrimination improvement (IDI) were computed to assess the improvement in risk stratification provided by the USPN model. Precision-Recall (PR) Curve, Given the imbalanced nature of the dataset (NEC cases vs. controls), the PR curve was employed to evaluate model performance. The area under the PR curve (AUC-PR) was calculated to provide a more robust assessment of predictive accuracy in the context of class imbalance. Statistical significance was defined as *p* < 0.05. All statistical analyses were performed using Python 3.2 and R 4.1.2.

### 2.9 Explainable AI (XAI) analysis

To gain insights into the factors driving model predictions, we employed SHAP (SHapley Additive exPlanations) values to quantify the contribution of each feature to the model's output. Feature importance was assessed based on the mean absolute SHAP values.

## 3 Result

### 3.1 Base line of all patients

Baseline characteristics of the study population are presented in Table 1. The cohort consisted of 191 neonates, 50 patients were diagnosed with NEC, divided into a training set (*n* = 134) and a validation set (*n* = 57). Necrotizing enterocolitis (NEC) was present in 26.2% of the overall cohort, with similar proportions in the training (26.1%) and validation (26.3%) sets. The majority of neonates presented with bowel edema (76.4% overall), with a slightly higher prevalence in the validation set (78.9%) compared to the training set (75.4%), although this difference was not statistically significant (*p* = 0.73). Most neonates exhibited normal bowel peristalsis (79.6% overall), with a slightly lower proportion in the validation set (77.2%) compared to the training set (80.6%). The cohort was slightly skewed towards males (55.0%), with a similar distribution in the training set (53.0% male) and a slightly higher proportion of males in the validation set (59.6%). The mean age of the neonates was 32.0 ± 4.4 days, with the training set having a slightly older mean age (32.3 ± 4.7 days) than the validation set (31.5 ± 3.7 days). The mean onset day of symptoms was 8.7 ± 8.3 days overall. The validation set had a slightly later mean onset day (9.2 ± 7.3 days) compared to the training set (8.4 ± 8.7 days). Mean bowel thickness was 2.2 ± 1.1 mm, with very similar values in both the training (2.2 ± 1.1 mm) and validation (2.1 ± 1.0 mm) sets. No statistically significant differences were observed between the training and validation sets for any of the examined variables.

**Table 1:** Basic information of all patients.

Variable	Total	Training	Validation	Statistic	*P*_Value
OB (no)	60 (31.4%)	46 (34.3%)	14 (24.6%)	1.35	0.25
OB (yes)	131 (68.6%)	88 (65.7%)	43 (75.4%)		
Gas (no)	84 (44%)	58 (43.3%)	26 (45.6%)	0.02	0.89
Gas (yes)	107 (56%)	76 (56.7%)	31 (54.4%)		
Bowel edema(no)	45 (23.6%)	33 (24.6%)	12 (21.1%)	0.12	0.73
Bowel edema(yes)	146 (76.4%)	101 (75.4%)	45 (78.9%)		
Bowel peristalsis(weaken)	39 (20.4%)	26 (19.4%)	13 (22.8%)	0.11	0.74
Bowel peristalsis(normal)	152 (79.6%)	108 (80.6%)	44 (77.2%)		
Seroperitoneum(no)	168 (88%)	117 (87.3%)	51 (89.5%)	0.03	0.86
Seroperitoneum(yes)	23 (12%)	17 (12.7%)	6 (10.5%)		
Transfusion(no)	171 (89.5%)	119 (88.8%)	52 (91.2%)	0.06	0.81
Transfusion(yes)	20 (10.5%)	15 (11.2%)	5 (8.8%)		
Ventilation(no)	134 (70.2%)	96 (71.6%)	38 (66.7%)	0.26	0.61
Ventilation(yes)	57 (29.8%)	38 (28.4%)	19 (33.3%)		
Antibiotic(no)	29 (15.2%)	19 (14.2%)	10 (17.5%)	0.14	0.71
Antibiotic(yes)	162 (84.8%)	115 (85.8%)	47 (82.5%)		
NRDS(no)	179 (93.7%)	127 (94.8%)	52 (91.2%)	0.36	0.55
NRDS(yes)	12 (6.3%)	7 (5.2%)	5 (8.8%)		
PDA(no)	159 (83.2%)	115 (85.8%)	44 (77.2%)	1.56	0.21
PDA(yes)	32 (16.8%)	19 (14.2%)	13 (22.8%)		
Distress(no)	175 (91.6%)	124 (92.5%)	51 (89.5%)	0.17	0.68
Distress(yes)	16 (8.4%)	10 (7.5%)	6 (10.5%)		
Dirty(no)	181 (94.8%)	129 (96.3%)	52 (91.2%)	1.16	0.28
Dirty(yes)	10 (5.2%)	5 (3.7%)	5 (8.8%)		
Embryolemma(no)	155 (81.2%)	108 (80.6%)	47 (82.5%)	0.01	0.92
Embryolemma(yes)	36 (18.8%)	26 (19.4%)	10 (17.5%)		
Delivary(eutocia)	101 (52.9%)	69 (51.5%)	32 (56.1%)	0.19	0.67
Delivary(cesarean)	90 (47.1%)	65 (48.5%)	25 (43.9%)		
Motherdiabetes(no)	148 (77.5%)	102 (76.1%)	46 (80.7%)	0.25	0.61
Motherdiabetes(yes)	43 (22.5%)	32 (23.9%)	11 (19.3%)		
MotherHBP(no)	170 (89%)	118 (88.1%)	52 (91.2%)	0.15	0.7
MotherHBP(yes)	21 (11%)	16 (11.9%)	5 (8.8%)		
Placentalinflammation(no)	179 (93.7%)	126 (94%)	53 (93%)	0	1
Placentalinflammation(yes)	12 (6.3%)	8 (6%)	4 (7%)		
NEC(no)	141 (73.8%)	99 (73.9%)	42 (73.7%)	0	1
NEC(yes)	50 (26.2%)	35 (26.1%)	15 (26.3%)		
Sex (female)	86 (45.03%)	23 (12.04%)	63 (32.98%)	0	1
Sex (male)	105 (54.97%)	27 (14.14%)	78 (40.84%)		
Age	32.04 ± 4.39	32.25 ± 4.65	31.54 ± 3.7	0.92	0.34
Gestational	259.27 ± 21.51	259.34 ± 20.57	259.09 ± 23.77	0.08	0.77
Polyembryony	0.14 ± 0.35	0.14 ± 0.35	0.14 ± 0.35	0	0.98
Weight	2,729.19 ± 746.08	2,718.17 ± 728.71	2,755.11 ± 791.46	0.11	0.74
Onset day	8.66 ± 8.26	8.43 ± 8.65	9.21 ± 7.29	2.21	0.14
Bowel thickness	2.16 ± 1.05	2.17 ± 1.06	2.14 ± 1.02	0	0.99
crp	10.42 ± 24.38	9.84 ± 23.22	11.77 ± 27.07	1.03	0.31
alp	206.29 ± 93.6	197.95 ± 88.29	225.91 ± 103.24	3.95	0.05
alb	34.12 ± 4.07	34.05 ± 4.23	34.27 ± 3.71	0.01	0.91
Procalcitonin	1.73 ± 5.03	1.82 ± 5.56	1.5 ± 3.51	1.51	0.22
plt	318.01 ± 105.35	319.28 ± 104.1	315.02 ± 109.12	0.01	0.92
inr	1.15 ± 0.14	1.14 ± 0.14	1.15 ± 0.13	0.04	0.85
wbc	12.82 ± 5.37	12.76 ± 5.3	12.97 ± 5.58	0.1	0.75
hgb	169.79 ± 26.74	170.37 ± 27.4	168.42 ± 25.32	0.03	0.86

### 3.2 Comparison of different machine learning methods

The performance of various machine learning algorithms in predicting NEC was evaluated using AUC and accuracy. The results are summarized in Table 2. Across the tested algorithms, XGBoost demonstrated the highest mean AUC (0.97, 95% CI: 0.92–0.99), indicating excellent discriminatory ability.

**Table 2:** Comparision of all algorithm.

Model	Mean AUC	AUC 95% CI Lower	AUC 95% CI Upper	Mean Accuracy
Logistic Regression	0.92	0.81	0.95	0.94
Random Forest	0.94	0.89	0.97	0.96
Gradient Boosting	0.94	0.89	0.97	0.94
SVC	0.94	0.89	0.96	0.92
Decision Tree	0.84	0.77	0.9	0.84
K-Nearest Neighbors	0.88	0.8	0.91	0.82
Naive Bayes	0.91	0.85	0.96	0.89
XGBoost	0.97	0.92	0.99	0.92
LightGBM	0.91	0.87	0.94	0.91
Ridge Classifier	0.91	0.85	0.95	0.92
Extra Trees	0.95	0.92	0.96	0.94
AdaBoost	0.92	0.9	0.95	0.93
Voting Classifier	0.92	0.9	0.97	0.93

### 3.3 Feature ranked and selection

**Figure 2:**
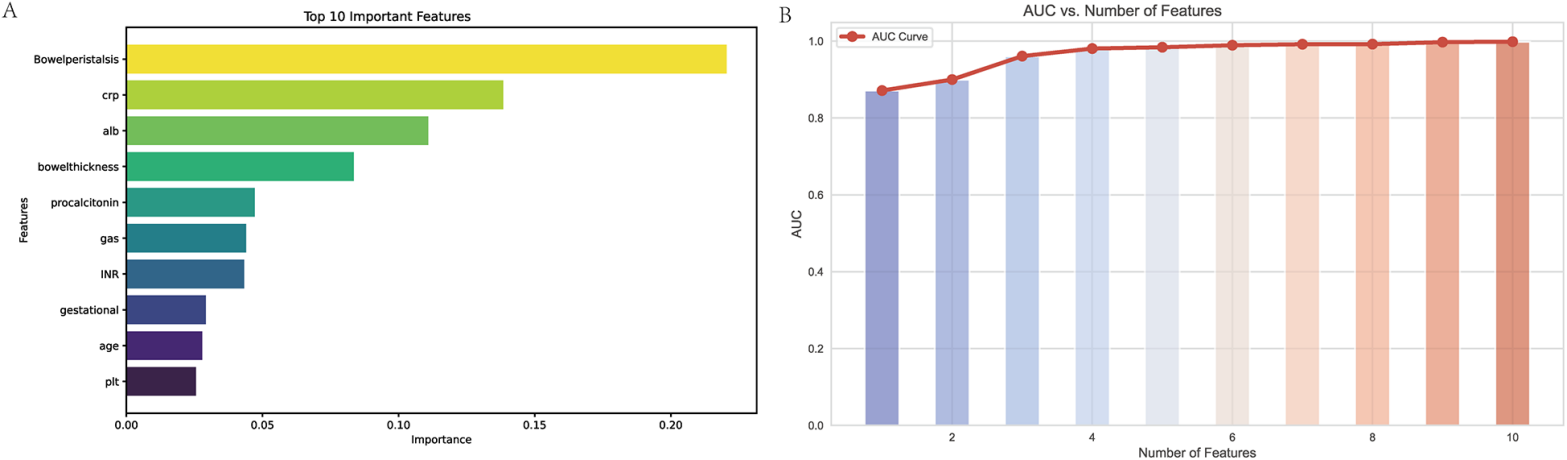
Variable importance and AUC performance analysis. **(A)** Top 10 Important Features: This bar chart displays the ten most important variables identified by the XGBoost model, ranked by their importance scores. The variable “Bowel peristalsis” is the most significant predictor, followed by C-reactive protein (CRP), albumin (ALB), and bowel thickness. The importance scores reflect the contribution of each feature to the model's predictive capability, with higher scores indicating greater relevance in predicting outcomes. **(B)** AUC vs. Number of Features: This line graph illustrates the relationship between the number of features used in the model and the corresponding Area Under the Curve (AUC) values. The AUC values increase with the addition of features, demonstrating improved predictive performance. Notably, the model achieves a robust AUC with just five features, indicating that a streamlined model can maintain high accuracy while simplifying the predictive process. The red line represents the AUC curve, with data points indicating the AUC values for each subset of features.

Through the application of the XGBoost algorithm, the top 10 most influential variables were identified based on their feature importance scores (Figure 2A). The relationship between the number of variables and the model's AUC was systematically evaluated to determine the optimal subset of features (Figure 2B). The analysis demonstrated that incorporating the top 5 variables achieved a robust AUC, with minimal incremental improvement observed when additional variables were included.

### 3.4 ROC curve

**Figure 3:**
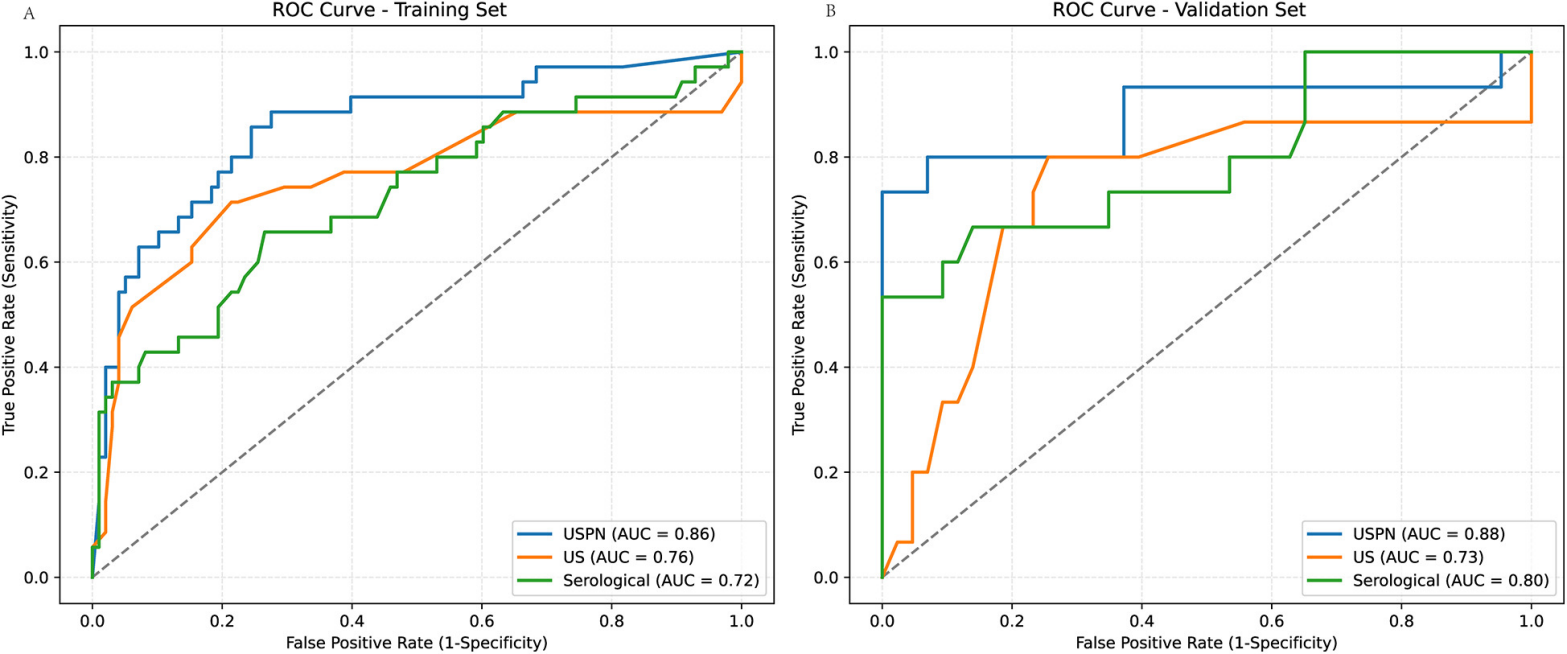
ROC curves for predictive models in training and validation sets. **(A)** ROC curves for the training set, comparing the USPN model (AUC = 0.86), US model (AUC = 0.76), and Serological model (AUC = 0.72). **(B)** ROC curves for the validation set, comparing the USPN model (AUC = 0.88), US model (AUC = 0.73), and Serological model (AUC = 0.80). The USPN model consistently demonstrates superior discriminatory performance compared to the US and Serological models in both training and validation datasets.

The predictive performance of the three models—the USPN model, US model, and Serological model—was assessed using ROC curve analysis. Figure 3 displays the ROC curves for each model in both the training (Figure 3A) and validation (Figure 3B) sets. Table 3 presents a comprehensive comparison of the USPN, US, and serological models in both the training and validation sets, including AUC, sensitivity, specificity, PPV, NPV, and accuracy. In the training set, the USPN model demonstrated the highest AUC (0.85), followed by the US model (0.76) and the serological model (0.72). The USPN model also exhibited a good balance between sensitivity (0.80) and specificity (0.77). In the validation set, the USPN model again achieved the highest AUC (0.88), with a 95% confidence interval suggesting robust performance (0.76–0.99). Notably, the USPN model in the validation set demonstrated perfect specificity (1.00) and PPV (1.00), along with high sensitivity (0.73), NPV (0.91) and accuracy (0.93).

**Table 3:** Different model comparision in train and test set.

Dataset	Model	AUC	AUC 95% CI Lower	AUC 95% CI Upper	Sensitivity	Specificity	PPV	NPV	Accuracy
Train	USPN	0.85	0.77	0.94	0.8	0.77	0.56	0.91	0.78
	US	0.76	0.65	0.86	0.51	0.93	0.75	0.84	0.82
	Serological	0.72	0.61	0.82	0.31	0.97	0.84	0.8	0.80
Test	USPN	0.88	0.76	0.99	0.73	1	1	0.91	0.93
	US	0.73	0.57	0.75	0.33	0.88	0.5	0.79	0.74
	Serological	0.75	0.65	0.85	0.4	1	1	0.82	0.84

### 3.5 Calibration curve

**Figure 4:**
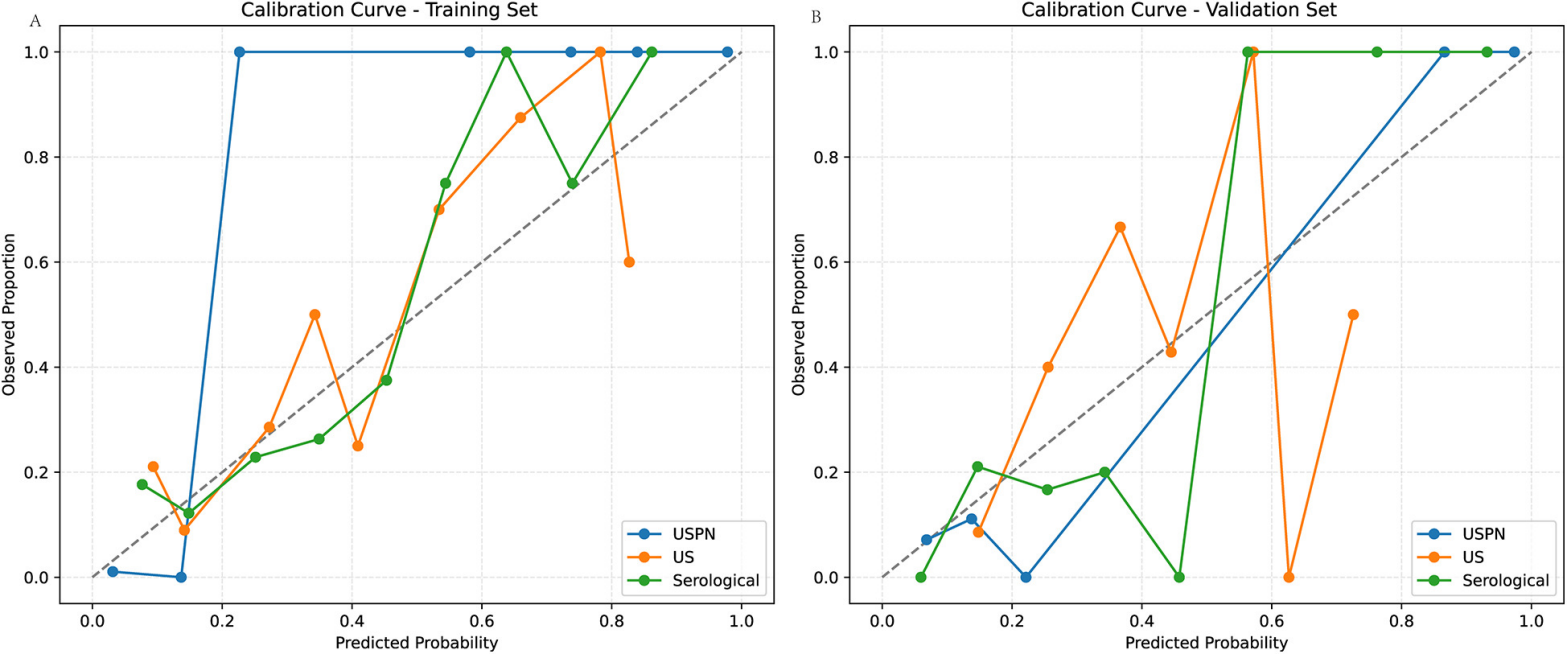
Calibration curves in the training and validation sets. **(A)** shows the calibration curves of the USPN, US, and Serological models in the training set. The USPN model (blue) generally demonstrates the closest alignment to the diagonal across most probability ranges, although it slightly overestimates risk at lower predicted probabilities. By contrast, the US model (orange) shows moderate agreement with the diagonal at mid-range probabilities but deviates for higher values, reflecting some degree of miscalibration. The Serological model (green) exhibits relatively good calibration at moderate predicted probabilities but becomes less accurate at the extremes. **(B)** illustrates the corresponding calibration curves in the validation set. The USPN model again appears best calibrated overall, remaining relatively close to the diagonal. The US model displays notable fluctuations, particularly at higher predicted probabilities, while the Serological model shows an underestimation trend at mid-range probabilities but aligns well with the diagonal at higher ranges. These findings are consistent with the quantitative calibration metrics, indicating that the USPN model provides superior calibration across both datasets compared with the other two models.

Model calibration was assessed using Brier scores, Hosmer-Lemeshow (HL) tests, and calibration slopes/intercepts. The USPN model demonstrated good calibration on both training and validation sets. However, the US and Serological models showed evidence of miscalibration, particularly on the training sets, as indicated by significant HL *p*-values (*p* < 0.05) and/or slopes deviating from 1. The US model on the test set showed a particularly concerning slope of 0.41 (Table 4). Calibration curves for the USPN, US, and serological models were generated to assess the agreement between predicted probabilities and observed proportions in both the training (Figure 4A) and validation (Figure 4B) sets. Ideally, a perfectly calibrated model would follow the diagonal dashed line, indicating perfect agreement.

**Table 4:** Calibration indication in different model.

Dataset	Model	Brier Score	HL *p*-value	Calibration Slope	Calibration Intercept
Train	USPN	0.01	0.65	1.02	0.02
	US	0.14	0.02	1.18	0.04
	Serological	0.16	0.04	1.22	−0.03
Test	USPN	0.06	0.6	1.11	−0.09
	US	0.16	0.05	0.41	0.26
	Serological	0.12	0.04	1.23	−0.12

### 3.6 Decision curve analysis (DCA)

**Figure 5:**
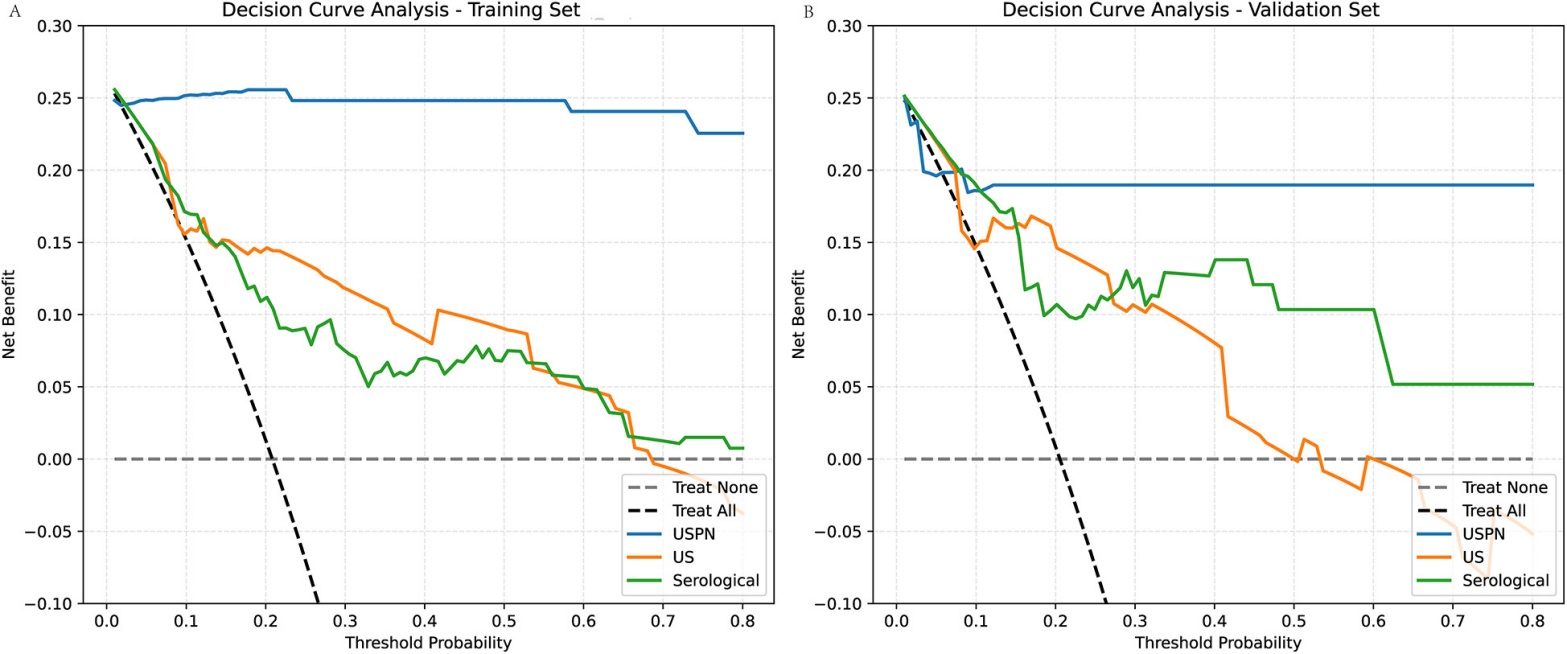
Decision curve analysis (DCA) for the training and validation sets. **(A)** For the training set compares the clinical net benefits of the USPN, US, and Serological models. The DCA shows the net benefit of each model across different threshold probabilities. The USPN model (blue line) provides the highest net benefit at most threshold probabilities, demonstrating superior clinical utility compared to the other models. The US model (orange line) shows a lower net benefit, particularly at higher threshold probabilities, reflecting its relatively poorer performance. The Serological model (green line) performs similarly to the US model, offering limited net benefit across most threshold probabilities. **(B)** For the validation set presents similar trends. The USPN model continues to outperform the other models across a wide range of threshold probabilities, showing the highest net benefit, particularly at threshold values between 0.1 and 0.5. The US model and Serological model again display lower net benefits, with the US model performing slightly better than the Serological model at certain points but still underperforming compared to the USPN model.

To evaluate the clinical utility of the USPN, US, and serological models, we performed DCA. Figure 5 presents the DCA curves for the training (Figure 5A) and validation (Figure 5B) sets. To further quantify the improvements in risk prediction offered by the USPN model, we calculated the NRI, IDI, and Relative Net Benefit, comparing the USPN model to both the US and serological models. These results are presented in Table 5. In the training set, the USPN model showed substantial improvements in risk classification compared to both the US (NRI = 0.39, IDI = 0.62) and serological (NRI = 0.60, IDI = 0.69) models. However, the relative net benefit was 0.00 in both comparisons in the training set. In the validation set, the USPN model continued to demonstrate improvements in risk prediction compared to the US (NRI = 0.28, IDI = 0.49) and serological (NRI = 0.33, IDI = 0.44) models. Importantly, in the validation set, the USPN model also showed a positive relative net benefit compared to both the US model (0.18) and the serological model (0.17). These results indicate that the USPN model not only improves risk classification but also provides a clinically meaningful net benefit compared to the other models in an independent validation set.

**Table 5:** DCA indication in different model.

Dataset	Model	NRI	IDI	Relative Net Benefit
Train	USPN vs. US	0.39	0.62	0.00
	USPN vs. Serological	0.60	0.69	0.00
Test	USPN vs. US	0.28	0.49	0.18
	USPN vs. Serological	0.33	0.44	0.17

### 3.7 Precision-recall (PR) curves

**Figure 6:**
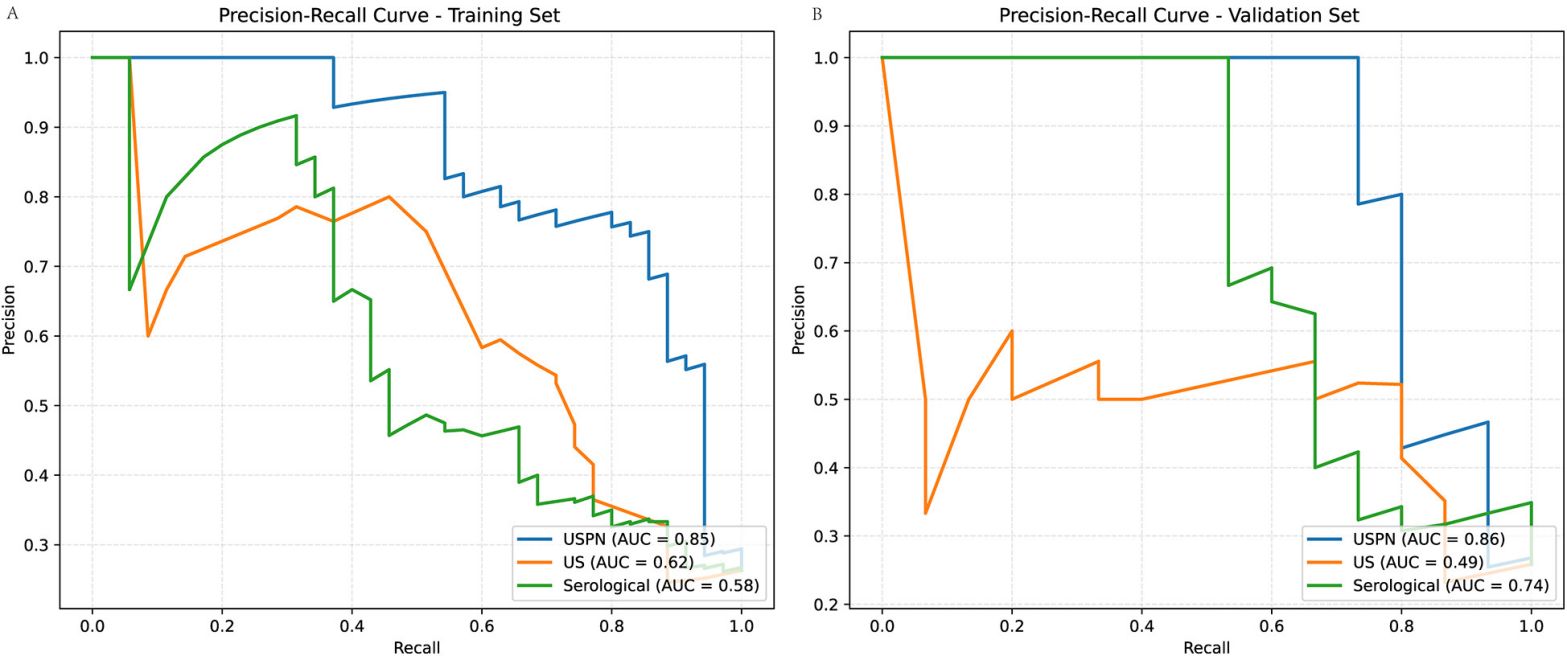
Precision-Recall (PR) curves for training and validation sets. **(A)** (Training Set): The USPN method (blue curve) achieves the highest AUC (0.85), followed by the US method (orange curve, AUC = 0.62) and the Serological method (green curve, AUC = 0.58). **(B)** (Validation Set): The USPN method maintains the highest AUC (0.86), while the US method (AUC = 0.49) and the Serological method (AUC = 0.74) exhibit lower performance.

To further evaluate the performance of the USPN, US and the serological model, we generated PR curves. Figure 6 presents the PR curves for both the training (Figure 6A) and validation (Figure 6B) set, these results emphasize the USPN model as the most robust and reliable model across both datasets, offering superior precision and recall compared to the US and Serological models.

### 3.8 Shapley additive exPlanations (SHAP)

**Figure 7:**
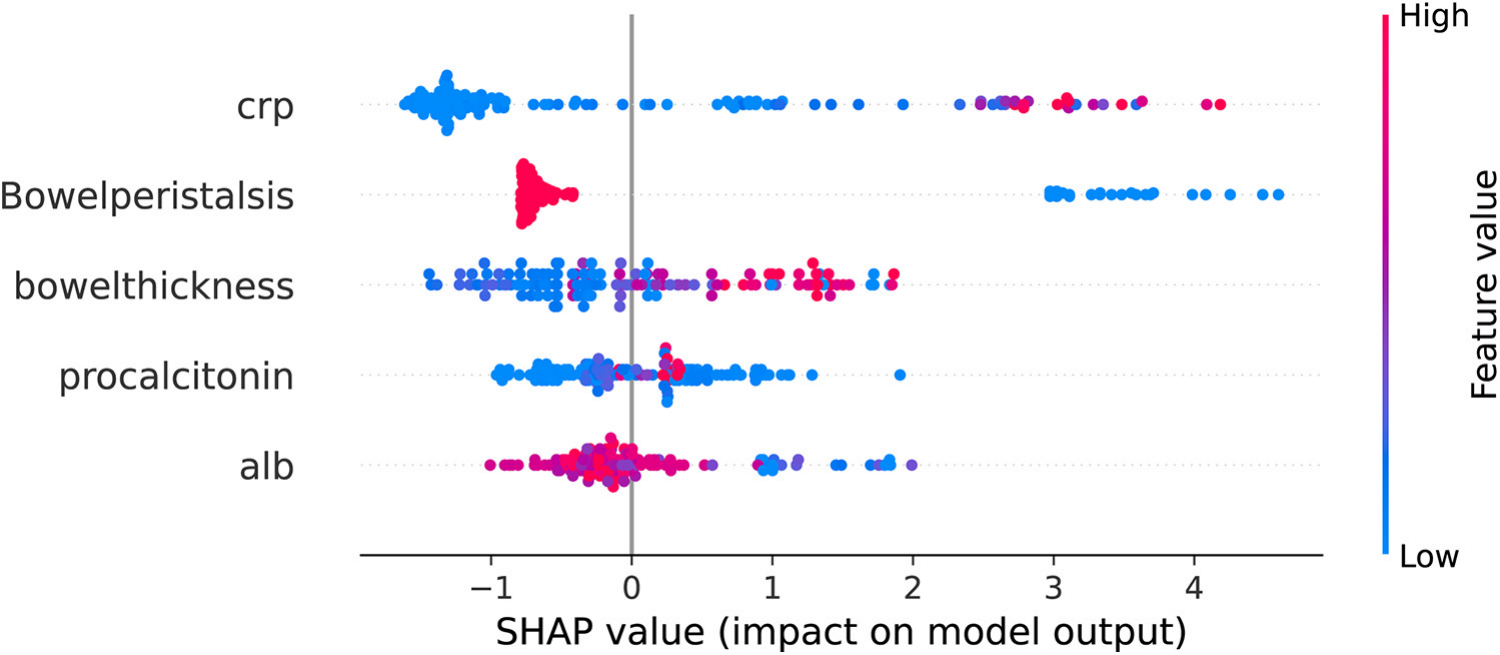
Beeswarm plot of SHAP values for top predictive features from XGBoost model. The beeswarm plot summarizes the SHAP values for the top five predictive features as determined by the XGBoost model. Each point on the plot represents a single patient. The *x*-axis denotes the SHAP value, representing the impact of the feature on the model's output (log-odds scale). Features are listed on the *y*-axis in descending order of importance. Color denotes the feature value for each patient, ranging from low (blue) to high (red), as indicated by the color gradient on the right. Positive SHAP values indicate that the feature contributes to increasing the predicted probability of NEC, while negative SHAP values indicate a contribution towards decreasing the predicted probability. The features displayed are: C-reactive protein, Bowel peristalsis, Bowel thickness, Procalcitonin, and Albumin.

The SHAP value plot (beeswarm) (Figure 7) was used to visualize the impact of different features (CRP, bowel peristalsis, bowel thickness, procalcitonin, and albumin) on the model's predictions. SHAP values quantify the contribution of each feature to the model's output, with positive values indicating an increase in the predicted outcome and negative values indicating a decrease. To enhance the clinical applicability of the USPN model, we analyzed the distribution of SHAP values for the top five features. The results indicated consistent NEC risk elevation when specific thresholds were crossed. Specifically, CR*P* > 20 mg/L, procalcitonin > 2.0 ng/ml, albumin < 25 g/L, bowel wall thickness > 2.6 mm, and absent or markedly reduced bowel peristalsis were associated with higher predicted risk of NEC. These findings offer potential clinical guidance for early risk stratification.

### 3.9 Waterfall plots illustrating feature contributions to model predictions

**Figure 8:**
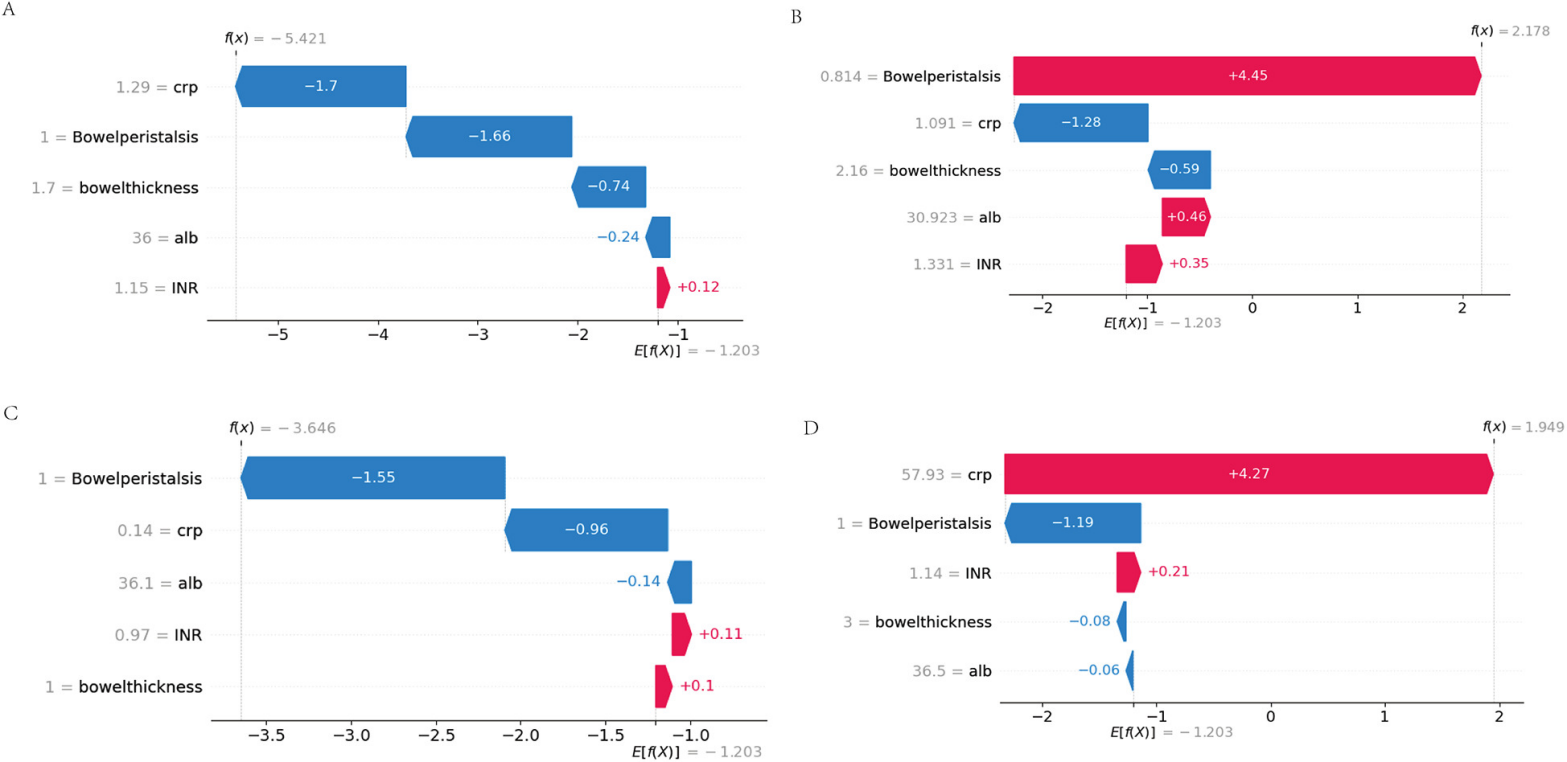
Waterfall plots of feature contributions across prediction-outcome categories. **(A)** True Negatives (TN): Feature contributions (negative SHAP values) dominated by physiological CRP (<5 mg/L) and normal peristalsis (≥3 episodes/hour). **(B)** False Positives (FP): Misclassification driven by transient CRP spikes (10–15 mg/L) overriding protective peristalsis signals. **(C)** False Negatives (FN): Early-stage NEC cases where moderate biomarker elevations failed to offset borderline peristalsis. **(D)** True Positives (TP): Synergistic contributions from hyperinflammation (CRP >20 mg/L), ileus (peristalsis = 0), and severe hypoalbuminemia (<2.0 g/dl).

To dissect the individual predictions generated by the XGBoost model, we generated waterfall plots using SHAP values, as presented in Figure 8. This figure showcases four representative cases, stratified by the concordance between actual and predicted outcomes.

### 3.10 Case-specific interpretation of false positive and false negative predictions

To further explore the clinical relevance of model errors, we analyzed the representative false positive (FP) and false negative (FN) cases shown in Figures 8B,C.

In the FP case, the model predicted NEC primarily due to markedly reduced bowel peristalsis (SHAP +4.45), despite low CRP (1.09 mg/L, SHAP −1.28) and normal bowel wall thickness (2.16 mm, SHAP −0.59). Clinical review revealed that the patient had early-onset sepsis with transient ileus, which likely accounted for the peristalsis suppression without actual NEC. This illustrates how the model may over-rely on a single dominant feature, leading to overestimation in the absence of supporting inflammation.

In the FN case, the model failed to predict NEC in an extremely preterm neonate with early-stage disease. SHAP analysis showed that normal peristalsis (SHAP −1.55) and very low CRP (0.14 mg/L, SHAP −0.96) significantly suppressed the predicted probability. Although NEC developed later, the patient's initial clinical profile was subtle, without marked inflammation or imaging changes. This case highlights the difficulty of early NEC detection when signs are not yet pronounced.

These observations emphasize the need for incorporating temporal biomarker trends, gestational age, and sepsis status into future models to improve performance in borderline or atypical cases.

## 4 Discussion

This study demonstrates that an XGBoost-based fusion model incorporating ultrasound and serological markers (USPN model) significantly improves the prediction of NEC in neonates compared to models relying solely on ultrasound or serological data. This finding highlights the potential of integrating multimodal data and machine learning to enhance diagnostic accuracy and inform clinical decision-making in this vulnerable population. The superior performance of the fusion model suggests that NEC is a complex disease process that is best characterized by a combination of imaging and biomarker data.

Our results build upon previous research that has explored the use of ultrasound or serological markers for NEC prediction. For example, Wang et al. retrospectively analyzed 144 neonates with suspected or confirmed NEC, comparing abdominal ultrasound and plain x-rays for diagnostic accuracy and prognostication. Their study found that ultrasound was superior to x-ray in detecting portal venous gas and intestinal dilatation in confirmed NEC cases. Furthermore, ultrasound findings of intestinal dilatation, bowel wall thickening, and ascites were significantly associated with the need for surgery or death, suggesting their potential utility in predicting disease severity. While this study highlights the value of abdominal ultrasound, it did not incorporate serological markers, limiting its ability to leverage the combined predictive power of both modalities, as explored in our current research (17). In contrast to studies focusing solely on imaging, Garg et al. (18) investigated the clinical impact of NEC-associated sepsis and its relationship to inflammatory markers. They found that infants with NEC-associated sepsis had significantly higher CRP levels and lower platelet counts at NEC onset and 24 h after onset compared to those without sepsis. While Garg et al. primarily focused on the *consequences* of NEC-associated sepsis and its impact on outcomes like length of stay and mortality, our study aims to leverage serological markers, alongside ultrasound findings, for *early prediction* of NEC development, whereas our study demonstrates the synergistic effect of combining these modalities within a machine learning framework.

Several studies have also explored the use of machine learning for NEC prediction. Leiva et al. (15) systematically reviewed the potential of multi-omics (genomics, proteomics) combined with machine learning to identify NEC biomarkers, highlighting its ability to decode disease subtypes and therapeutic targets through heterogeneous data integration. However, their analysis revealed critical limitations in existing approaches: (1) reliance on single-omics data that may not fully capture the complexity of NEC; (2) small sample sizes in many studies, potentially leading to overfitting risks; (3) exclusion of imaging modalities like ultrasound, which limits early diagnostic utility. In contrast, our study advances the field by addressing these gaps. We rigorously compared 12 machine learning algorithms to optimize model generalizability, ultimately selecting XGBoost model with SHAP-based interpretability. Crucially, we integrated ultrasonographic markers with serological markers. This multimodal strategy not only improves sensitivity for early NEC but also addresses the “black-box”. By bridging imaging biomarkers with host response dynamics, we provide a clinically actionable tool that aligns with Leiva's call for “phenotype-aware AI models” while overcoming the translational barriers of pure omics approaches.

Although the predictive variables were collected within 24 h before the formal diagnosis of NEC, they reflect routine monitoring performed during the early phase of clinical suspicion, prior to overt disease recognition or intervention. The model was specifically designed to operate at this early stage, utilizing parameters triggered by subtle signs rather than clear NEC manifestations.

Elevated CRP and procalcitonin levels, indicative of systemic inflammation, were strong predictors of NEC in our study, consistent with the established role of inflammation in the pathogenesis of the disease. Zeng's (19). study demonstrate the similar result. Gaudin et al. (20) further emphasized the prognostic value of CRP, demonstrating a correlation between elevated CRP levels and the risk of post-NEC intestinal stricture. our findings, combined with Gaudin et al.'s work, highlight the continued clinical relevance of CRP, particularly in assessing disease severity and predicting long-term complications. The readily availability and widespread use of CRP and procalcitonin assays make it a valuable tool in the initial assessment of NEC risk, Lee's (21) findings were similar with ours.

Our study identified hypoalbuminemia as a significant risk factor for the development of NEC, consistent with the findings of Mohd Amin et al., (22) who demonstrated that a low albumin level, particularly when combined with elevated CRP (CRP/ALB ratio ≥ 3), is strongly associated with poor outcomes, including the need for surgical intervention and mortality in neonates with NEC. Hypoalbuminemia may reflect systemic inflammation, malnutrition, or increased vascular permeability, all of which are implicated in the pathogenesis of NEC. The liver's reduced capacity to synthesize albumin in preterm infants, compounded by the inflammatory response, may further exacerbate this condition. The CRP/ALB ratio, as highlighted by Mohd Amin et al., serves as a valuable prognostic tool, integrating both inflammatory and nutritional status, which aligns with our findings that hypoalbuminemia independently predicts NEC risk.

Reduced bowel peristalsis and increased bowel wall thickness, as assessed by ultrasound, reflect intestinal ischemia and inflammation, key features of NEC, Chen demonstrated that in patients with reduced or absent intestinal peristalsis, the incidence of NEC is ten times higher than in those with normal peristalsis (23). This finding is consistent with our study, which also identified reduced bowel peristalsis as an independent risk factor for NEC. Esposito et al., (24) in their comprehensive review of NEC imaging, further support the significance of these ultrasound findings. They highlight that in the early stages of NEC, when x-ray findings may be non-specific, ultrasound can reveal direct signs such as bowel wall thickening (generally considered pathological when exceeding 2.6 mm) and abnormal bowel wall echoic patterns, reflecting the loss of normal wall layering due to inflammation and edema. Our study's identification of increased bowel wall thickness as an independent risk factor aligns with this observation, reinforcing the value of ultrasound in detecting early intestinal changes indicative of NEC. Priyadarshi's findings are also similar to ours (25).

While these ultrasound-based features are strongly associated with NEC risk, analysis of misclassified cases revealed that their predictive performance may vary depending on the broader clinical context. In certain cases, reduced bowel peristalsis alone contributed disproportionately to high-risk predictions, even when inflammatory markers such as CRP remained low and bowel wall thickness was within normal range. These false positive predictions often occurred in neonates with transient ileus or non-NEC-related sepsis, suggesting that peristalsis, though sensitive, may lack specificity when interpreted in isolation.

Conversely, false negative predictions were more frequently observed in extremely preterm infants with early-stage or atypical NEC. In these cases, CRP and procalcitonin levels were often within normal limits, and bowel ultrasound findings were subtle or absent. Such presentations, while clinically recognized, may escape detection in models that rely solely on static, single-timepoint data.

These findings underscore the need to incorporate additional contextual and temporal information into future model iterations. Potential strategies include the use of time-series trends in inflammatory biomarkers, integration of gestational age and birth weight, and inclusion of comorbid conditions such as sepsis or hemodynamic instability. Furthermore, modeling feature interactions—such as interpreting reduced peristalsis as high risk only in the presence of elevated CRP—may enhance model specificity and reduce misclassification in borderline clinical scenarios.

In our cohort, only 13 neonates (6.8%) had a gestational age <32 weeks, which limited the feasibility of performing gestational-age–stratified analysis of ultrasound findings. Future multicenter studies with larger and more balanced cohorts are needed to investigate how NEC presentation varies with gestational maturity, as highlighted by Battersby et al. (26).

## 5 Limitations

This study has several notable limitations. First, the retrospective design introduces potential selection and information biases, as data were extracted from existing medical records rather than prospectively collected. Although we implemented strict inclusion and exclusion criteria, residual confounding may still exist. Second, this was a single-center study conducted in a tertiary NICU, which may limit the diversity of patient populations, clinical practices, and imaging protocols—factors that can affect model generalizability. Third, the relatively small sample size (*n* = 191), while sufficient for initial model development and internal validation, increases the risk of overfitting and may not fully capture the heterogeneity of NEC presentations.

In fact, the discrepancy between the training AUC (0.97) and validation AUC (0.88) may reflect some degree of overfitting, despite the implementation of mitigation strategies such as 10-fold cross-validation, hyperparameter tuning via grid search, and SHAP-based feature selection. These techniques helped reduce dimensionality and overfitting risk, but further refinement is still necessary.

To improve the robustness and external applicability of the USPN model, future studies should focus on prospective validation in larger, multicenter cohorts. This would allow model calibration across varying institutions and patient subgroups, enhancing its clinical utility. In addition, the incorporation of further regularization techniques (e.g., L1/L2 penalty), model simplification, or ensemble averaging may help improve performance stability and reduce the risk of overfitting in future implementations.

## 6 Conclusion

In conclusion, our study demonstrates that an XGBoost-based fusion model incorporating ultrasound and serological markers significantly improves the prediction of NEC in neonates. This finding has important clinical implications and highlights the potential of integrating multimodal data and machine learning to enhance diagnostic accuracy and inform clinical decision-making in this vulnerable population. Future research should focus on validating our findings in larger multi-center studies and exploring the use of the model to guide clinical decision-making and to personalize treatment strategies.

## Data Availability

The datasets presented in this article are not readily available because of concerns regarding patient privacy and confidentiality. Requests to access the datasets should be directed to the corresponding author via email: feitianlu.fpm@163.com.
